# Linking the oral microbiome and salivary cytokine abundance to circadian oscillations

**DOI:** 10.1038/s41598-021-81420-3

**Published:** 2021-01-29

**Authors:** Anujit Sarkar, Melanie N. Kuehl, Amy C. Alman, Brant R. Burkhardt

**Affiliations:** 1 College of Public Health, University of South Florida, Tampa, FL 33612 USA; 2 Department of Cell Biology, Microbiology and Molecular Biology, University of South Florida, Tampa, FL 33620 USA; 3 Present Address: IPS Labs, 1 Harvard Way, Hillsborough Township, NJ 08844 USA

**Keywords:** Microbial communities, Interleukins

## Abstract

Saliva has immense potential as a diagnostic fluid for identification and monitoring of several systemic diseases. Composition of the microbiome and inflammation has been associated and reflective of oral and overall health. In addition, the relative ease of collection of saliva further strengthens large-scale diagnostic purposes. However, the future clinical utility of saliva cannot be fully determined without a detailed examination of daily fluctuations that may occur within the oral microbiome and inflammation due to circadian rhythm. In this study, we explored the association between the salivary microbiome and the concentration of IL-1β, IL-6 and IL-8 in the saliva of 12 healthy adults over a period of 24 h by studying the 16S rRNA gene followed by negative binomial mixed model regression analysis. To determine the periodicity and oscillation patterns of both the oral microbiome and inflammation (represented by the cytokine levels), two of the twelve subjects were studied for three consecutive days. Our results indicate that the Operational Taxonomic Units (OTUs) belonging to *Prevotella*, SR1 and *Ruminococcaceae* are significantly associated to IL-1β while *Prevotella* and *Granulicatella* were associated with IL-8. Our findings have also revealed a periodicity of both the oral microbiome (OTUs) and inflammation (cytokine levels) with identifiable patterns between IL-1β and *Prevotella*, and IL-6 with *Prevotella*, *Neisseria* and *Porphyromonas*. We believe that this study represents the first measure and demonstration of simultaneous periodic fluctuations of cytokine levels and specific populations of the oral microbiome.

## Introduction

Saliva is a biofluid with much promise for identification of numerous disease biomarkers^1,2^. The use of saliva as a bio-reservoir for this purpose is also highly pragmatic. The relative ease of rapid collection with non-invasive accessibility and generous sample size (typically 1 ml) is extremely advantageous within a clinical setting especially if coupled to routine medical visits. Furthermore, the quantity of information that can be obtained from a saliva sample and utilized for analytical purposes is extensive. In examination of the saliva, large scale “omic” approaches, termed “salivaomics”, can be employed such as lipidomics, proteomics and metabolomics^3–5^. Single analyte determination can also be performed examining markers of inflammation, periodontal disease, and hormones such as cortisol^6,7^. Taken together, the relative abundance of measurable biomarkers in saliva has shown promise in the therapeutic identification of numerous diseases ranging from cancer^8–10^, Sjögren's syndrome^10^, type 1 and 2 diabetes mellitus^6,11^, psychiatric disorders^12^, cardiovascular disease^13^ and many others (reviewed in^1^).

In addition to the numerous proteins and metabolites present in the saliva, there is also an abundant amount of nucleic acids including DNA, mRNA and microRNA^14,15^. Approximately 30% of the DNA present in the saliva is of non-human origin and is from the oral microbiome. The composition of the oral microbiome is an extremely complex niche with estimates of up to 100 billion bacteria with 700 predominant taxa^16^. The oral microbiome composition can be impacted by numerous factors such as periodontal disease, diet or smoking^17,18^. The relationship between the oral microbiome and local and systemic diseases has been demonstrated and is still being actively investigated across various disciplines^19^. Various studies have indicated that the composition of the microbiome are strongly associated with changes in periodontal status and can be an indicator of systemic diseases^20,21^. In addition, the composition of the oral microbiome is believed to affect overall inflammation and immunology. For example, periodontal disease has been associated with cardiovascular disease, possibly due to the induction of chronic inflammation^22^. There is increasing evidence that specific oral bacterial species and particularly periodontal pathogens have an etiological role in atherogenesis which is associated with a heightened state of oral and systemic inflammation^23,24^. The oral microbiome and inflammation have been associated with numerous other diseases as well, such as cancer, Alzheimer’s, metabolic syndrome and mental illness^25^. Therefore, both the oral microbiome and oral inflammation could provide useful insights that may eventually lead to salivary diagnostics for the prediction and identification of various diseases.

Saliva’s use as a diagnostic fluid has been hindered due to a lack of understanding of biomolecules within saliva and their relevance to disease etiology, combined with the diurnal/circadian variations of these biomolecules^1^. If saliva is to be utilized as a non-invasive source of biomarkers indicating systemic disease, biological variation in association with diurnal rhythms must be considered and evaluated. At present, there have been limited studies examining the circadian oscillations of the human salivary microbiome and with conflicting conclusions^26,27^. Most studies examining diurnal microbiome variation have been performed evaluating the gut^28–31^. Similarly, limited studies have been conducted examining circadian rhythm of oral inflammation as determined by measurement of various cytokines^32^. Altogether, similar studies have examined diurnal variation within both the oral microbiome and various inflammatory biomarkers separately but none to our knowledge have examined these simultaneously and longitudinally within the same individuals.

To examine oral inflammatory and microbiome diurnal rhythms, we examined longitudinal saliva samples collected from 12 healthy adults and measured a panel of cytokines by multiplexing analysis and the oral microbiome via 16S rRNA analysis across both a 24-h time period and across 72 h for 2 individuals. Following determination of cytokine concentrations and bacterial populations, we examined inflammatory periodicity by performing a normalization method on the measured cytokines and the association between the cytokines and specific bacterial populations. We also tested the association between cytokine levels and OTUs from oral microbiome using a negative binomial mixed model regression to account for both the repeated measures taken on the same individuals over time and the over-dispersion commonly found in microbiome count data.

## Results

### Participant characteristics

Participant characteristics are displayed in Table 1. The mean participant age was 26.5 (± 5.2) years. The majority were female (58.3%) and White, non-Hispanic (83.3%). Average BMI was 23.1 (± 3.5) and most participants (11 out of 12) were in the normal (n = 6) to overweight (n = 5) BMI category. The average amount of sleep prior to first collection was 7.1 ± 0.79 h. All participants followed a typical sleep/wake cycle of daytime activity followed by nighttime sleep. The average wake time was 7:47 A.M. ± 1.3 S.D. and average time to bed was 12:17 A.M. ± 1.3 S.D. with a typical time of wake activity of 16 h Detailed listing of collection times is supplied in Supplementary Table S1. Salivary cotinine levels were also determined and found to be below 10 ng/ml for all participants. In addition, participants completed an oral health questionnaire inquiring about gingival condition and if loose tooth was present. None reported a loose tooth and 9 out of 12 reported gingival condition in the excellent, very good or good category with 3 reporting fair condition.

**Table 1:** Subject characteristics (n = 12).

Characteristics	
Age (years)*	26.5 (± 5.2)
Male (n)^†^	5 (41.7)
**Race/ethnicity (n)**^†^	
White	10 (83.3)
Hispanic	2 (16.7)
BMI (kg/m^2^)*	23.1 (± 3.5)
**BMI category (n)**^†^	
Underweight (< 18.5 kg/m^2^)^†^	1 (8.3)
Normal (18.5– < 25 kg/m^2^)^†^	6 (50)
Overweight (25– < 30 kg/m^2^)^†^	5 (41.7)
Obese (≥ 30 kg/m^2^)^†^	0 (0)
Current smoker (n; cotinine > 10 ng/ml)	0
Amount of sleep prior to collection (h)*	7.1 ± 0.79
Average wake time*	7:47 A.M. ± 1.3
Average time to bed*	12:17 A.M. ± 1.3

*Data presented as mean ± SD.

^†^Data presented as number (%).

### Alpha diversity and diurnal variation in composition of the salivary microbiome

**Figure 1:**
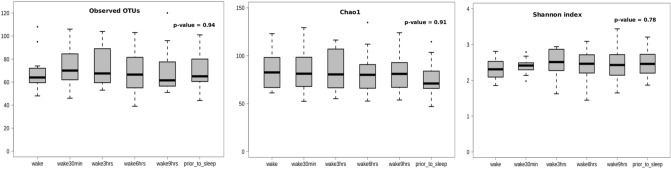
Alpha diversity analysis of oral microbiome obtained from longitudinally collected saliva samples from 12 individuals. Operational taxonomic units (*left panel*), Chao1 (*middle panel*), and Shannon Diversity Index analysis (*right panel*). For each of the metric (Observed OTUs, Chao1 and Shannon Index), Mann–Whitney test was used to determine if the values differ across the time-points (represented by each boxplot) and the corresponding p-values are shown at the top right of each plot.

**Figure 2:**
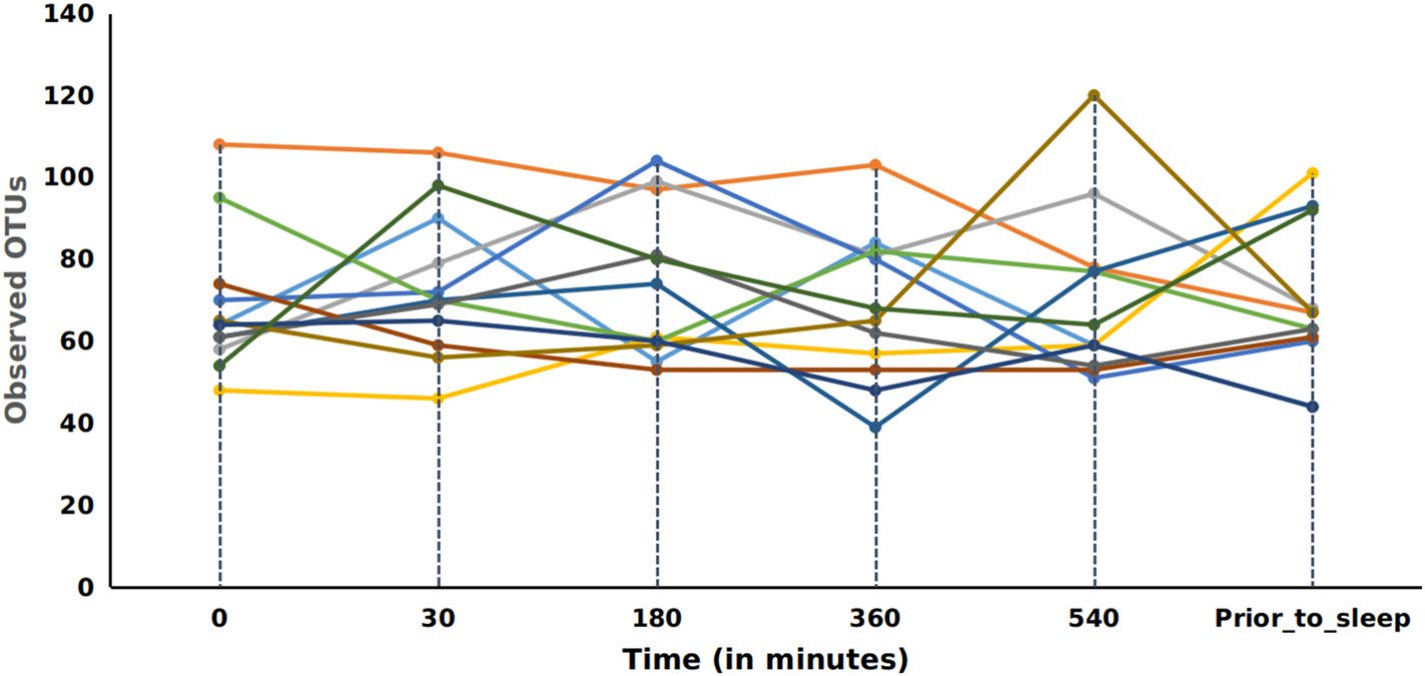
Observed Operational Taxonomic Units (OTUs) over time. OTUs are shown per subject as delineated by individual lines. X-axis units are shown as minutes post wake per individual.

**Figure 3:**
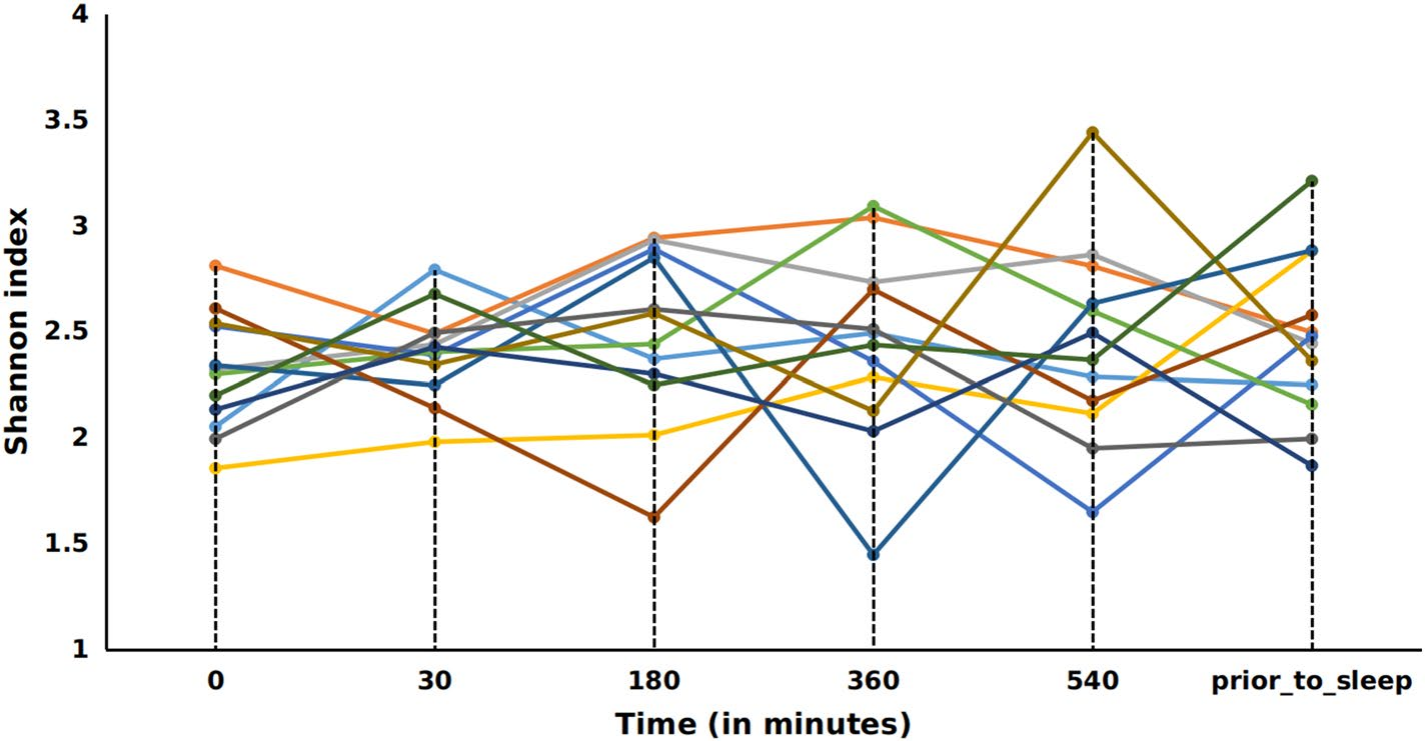
Shannon Diversity Index (richness and evenness) over time. OTUs are shown per subject as delineated by individual lines. X-axis units are shown as minutes post wake per individual.

**Figure 4:**
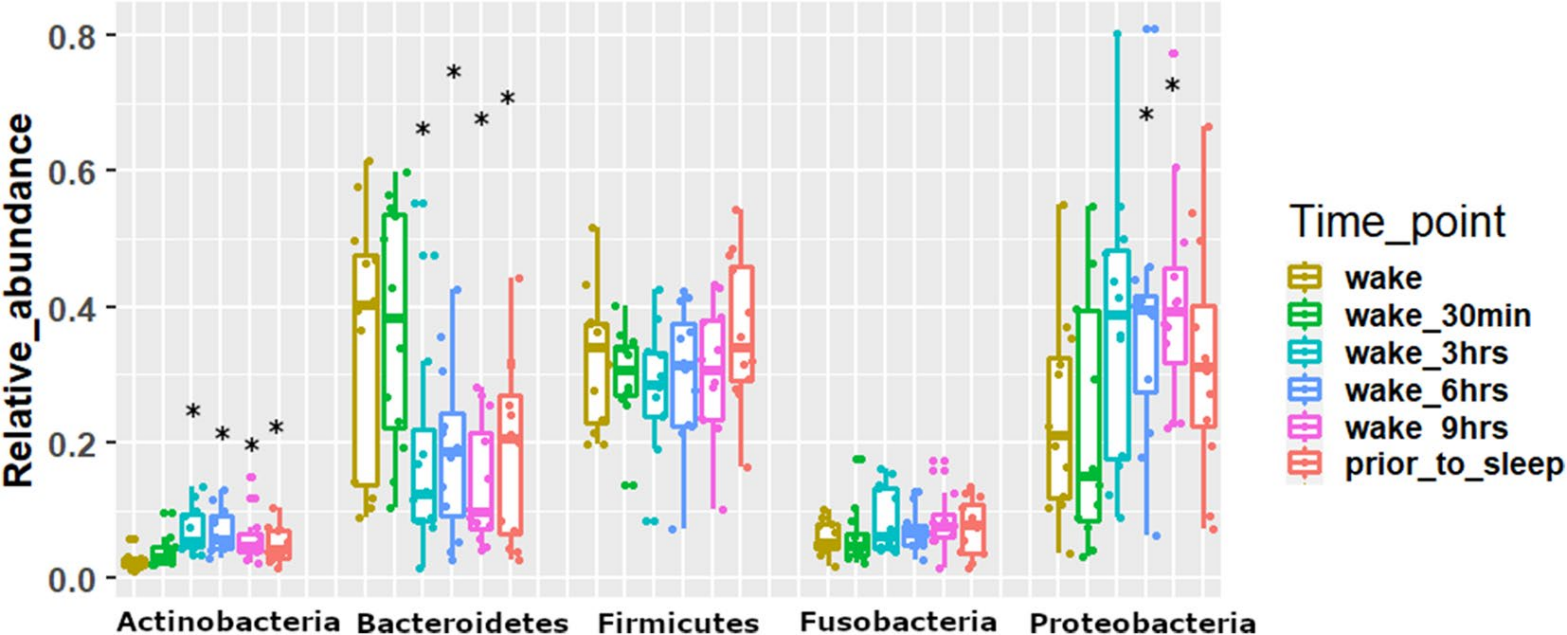
Taxonomic summary of relative abundance based on phyla abundance. X-axis represents the major bacterial phyla observed here while Y-axis represents the corresponding relative abundances. For each phylum, six plots have been made and each box represents a time point. Each boxplot has been made based on the relative abundance of the phylum for the 12 subjects. Mann–Whitney test was used to determine if the relative abundances of any phylum differ across the time-points (represented by color of each boxplot) with reference to the ‘wake’. Asterisks over a boxplot denote significant difference (increase or decrease) in relative phylum abundance in comparison to ‘wake’ time point.

**Figure 5:**
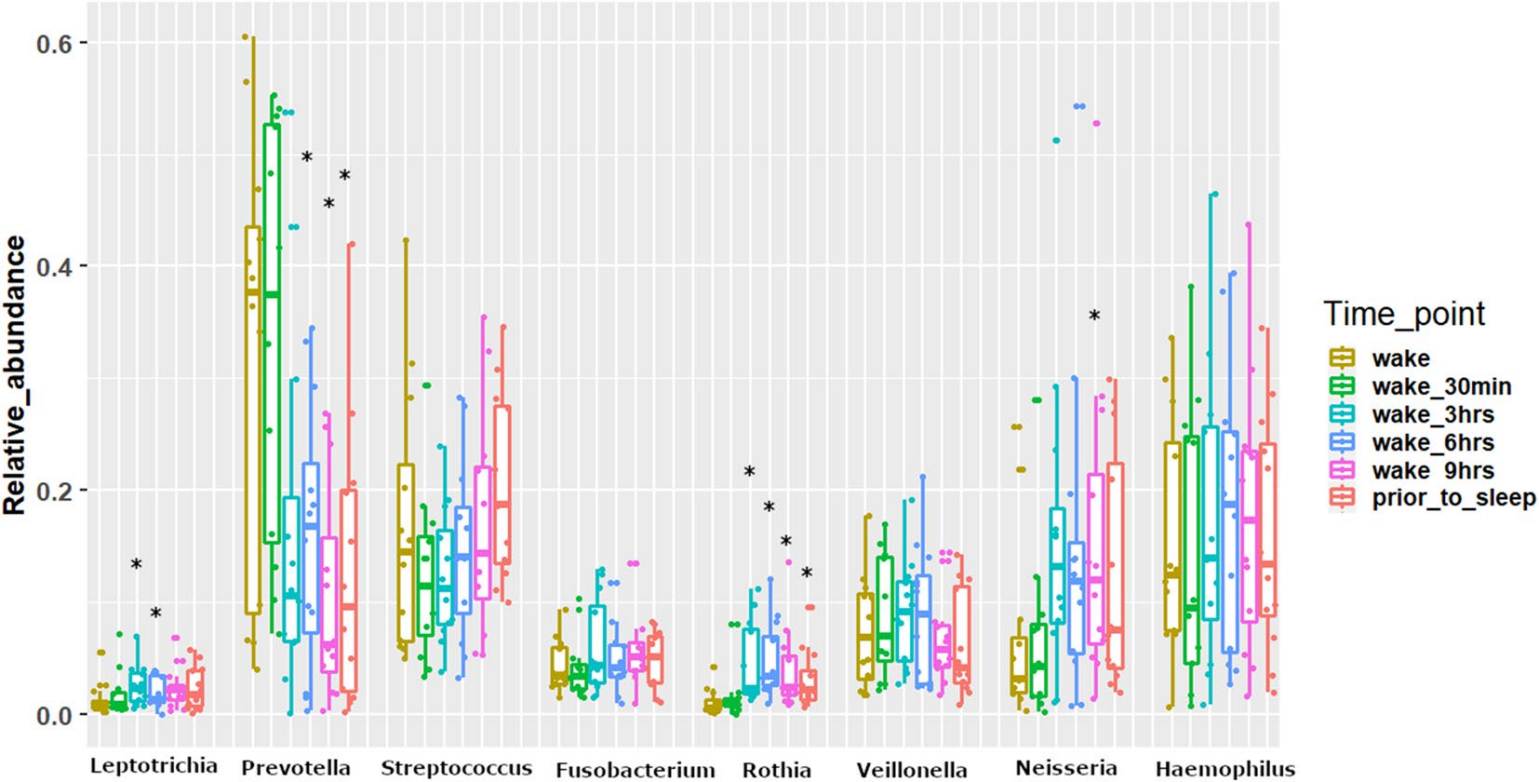
Taxonomic summary of relative abundance based on genera abundance. X-axis represents the major bacterial genera observed here while Y-axis represents the corresponding relative abundances. For each genus, six plots have been made and each box represents a time point. Each boxplot has been made based on the relative abundance of the genus for the 12 subjects. Mann–Whitney test was used to determine if the relative abundances of any genus differ across the time-points (represented by color of each boxplot) with reference to the ‘wake’. Asterisks over a boxplot denote significant difference (increase or decrease) in relative genus abundance in comparison to ‘wake’ time point.

Alpha diversity analysis was performed to measure the overall bacterial richness and composition within the salivary microbiome. Initial run metrics of the 16S Ilumina MiSeq analysis identified 223 operational taxonomic units (OTUs) with 7033 average reads per sample (data not shown). Observed OTUs were evaluated along with Shannon Diversity Index (SDI) and Chao1 analysis (Fig. 1) in relation to time of sample collection across the 12 subjects with samples collected at 6 time points (at waking, after 30 min., 3 h., 6 h., 9 h. and just prior to sleep) over a single 24 h period. The bacterial richness, diversity or abundance of the oral microbiome did not significantly differ within the samples among individuals either at matched time of collection or across time of collections (Fig. 1). In addition, no considerable change in the number of observed OTUs or SDI were observed across time for any of the 12 subjects (Figs. 2 and 3). We additionally measured changes in the relative abundance at both the phylum (Fig. 4) and genus level of the oral microbiome obtained from the saliva samples (Fig. 5). Major phylums identified included *Actinobacteria, Bacteroidetes, Firmicutes, Fusobacteria* and *Proteobacteria* (Fig. 4). We did not observe diurnal variation in phylum relative abundance of *Firmicutes* and *Fusobacteria*. However, compared to the relative abundance at waking, we did observe a significant increase in relative abundance of *Actinobacteria* and a significant decrease in relative abundance of *Bacteroidetes* at all time points, except for 30 min after waking (Fig. 4). The relative abundance of *Proteobacteria* was significantly increased at 6 and 9 h after waking and decreased approaching sleep (Fig. 4).

At the genus level (Fig. 5), major bacterial genera observed were *Fusobacterium, Haemophilus, Leptotrichia, Neisseria, Prevotella, Rothia, Streptococcus* and *Veillonella*. No significant changes were observed for genus relative abundance for *Fusobacterium, Streptococcus, Haemophilus* and *Veillonella*. Compared to relative abundance measured at wake, *Rothia* relative abundance significantly increased by 3 h after waking and remained significantly increased for the rest of the day, whereas a significant decrease in relative abundance was observed for *Prevotella* at 6 h after waking and all further collections. In addition, there was a significant increase of relative abundance of *Leoptotrichia* at wake + 3 and 6 h, and for *Neisseria* for wake + 9 h.

### Diurnal variation in concentration of salivary cytokines

**Figure 6:**
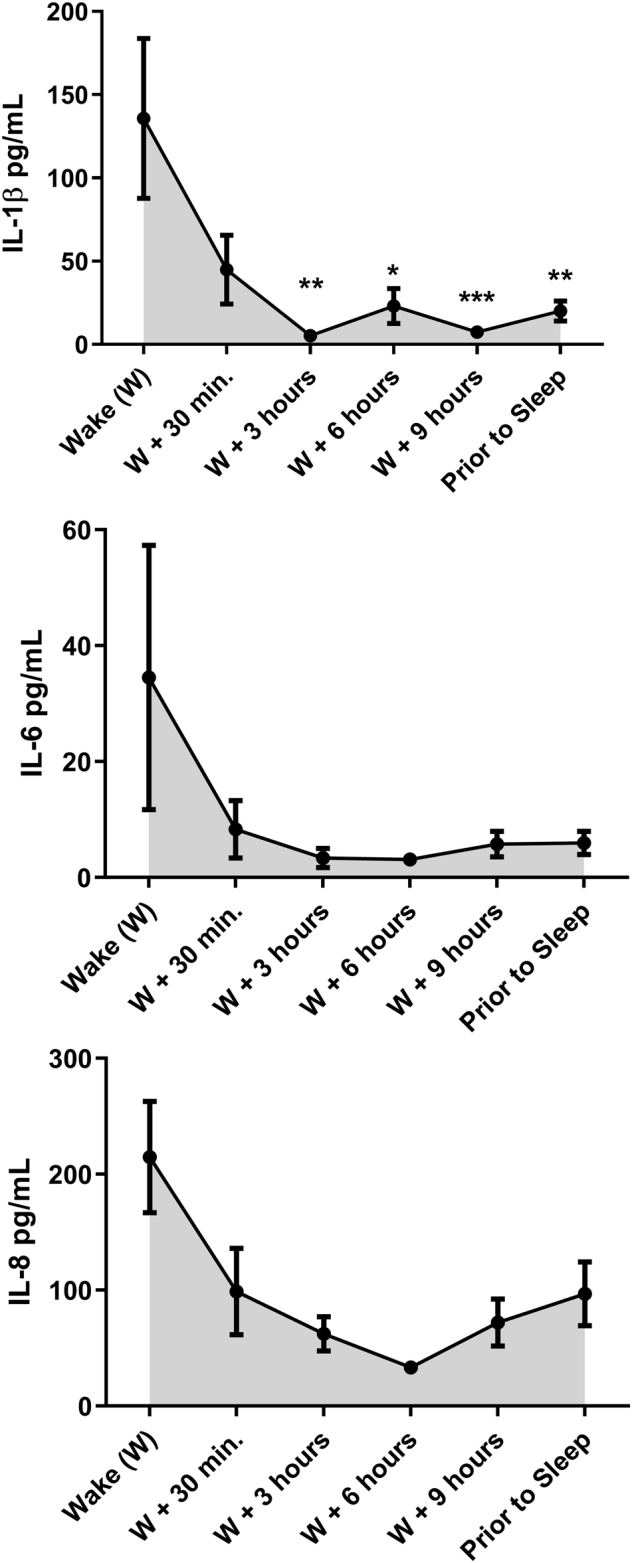
Cytokine concentration of saliva samples as determined by multiplexing analysis across 12 subjects. Data are shown as mean ± SE across subjects per time of collection for (**A**) IL-1β, (**B**) IL-6, and (**C**) IL-8. Asterisk denotes significant difference from cytokine concentration at wake, *p < 0.05, **p < 0.01, and **p < 0.001 as determined by repeated-measures ANOVA.

The general trend observed for all cytokines with regard to the 24-h temporal pattern was increased concentration at wake followed by a decrease within 30 min (Fig. 6). For IL-1β, decreases in concentration were statistically significant using repeated measures ANOVA for all subsequent timepoints except for 30 min after waking (Fig. 6A). Similar trends were observed for IL-6 and IL-8 but not found to be statistically significant (Fig. 6B,C).

### Association between cytokine levels and salivary microbiome

We examined the association of salivary microbiome OTUs (independent variable) and cytokine levels (dependent variable) using negative binomial mixed models specifying a random intercept and adjusted for age, sex, BMI, gum status, and the time of sample collection (Supplementary Table S2). We did not find any significant associations between any OTU and IL-6. For IL-8, an OTU belonging to the genus *Prevotella* (Prevotella6) and *Granulicatella* were negatively associated while *Aggregatibacter* and *Atopobium* were found to have a weak positive association. For IL-1β, OTUs belonging to *Prevotella* and *Ruminococcaccae* were positively associated, while an OTU representing SR1 showed negative association. Additionally, another OTU from *Prevotella* was positively associated while *Streptococcus* was negatively associated, although the associations were weak. Results are summarized in Supplementary Table 2.

### Periodicity of salivary microbiome and cytokine concentration

**Figure 7:**
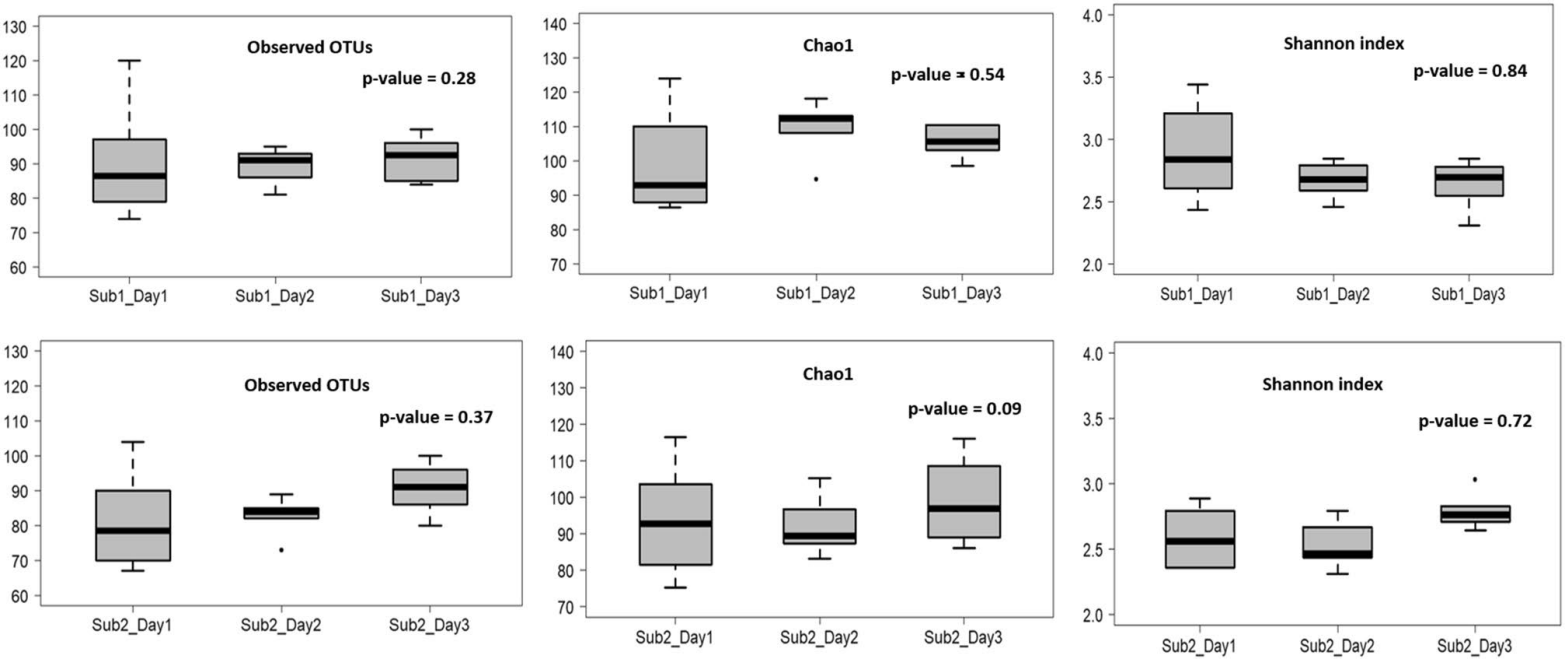
Alpha diversity analysis of oral microbiome for two subjects over three days. Operational taxonomic units (*left panel*), Chao1 (*middle panel*), and Shannon Diversity Index analysis (*right panel*). The upper panel represents the Subject1 while the lower panel represents the Subject2. For each metric (observed OTUs, Chao1 and Shannon index), three boxplots represents were drawn representing each day. Mann–Whitney test was used to determine if the values differ across the three days and p-value obtained have been noted on the top right of each plot.

**Figure 8:**
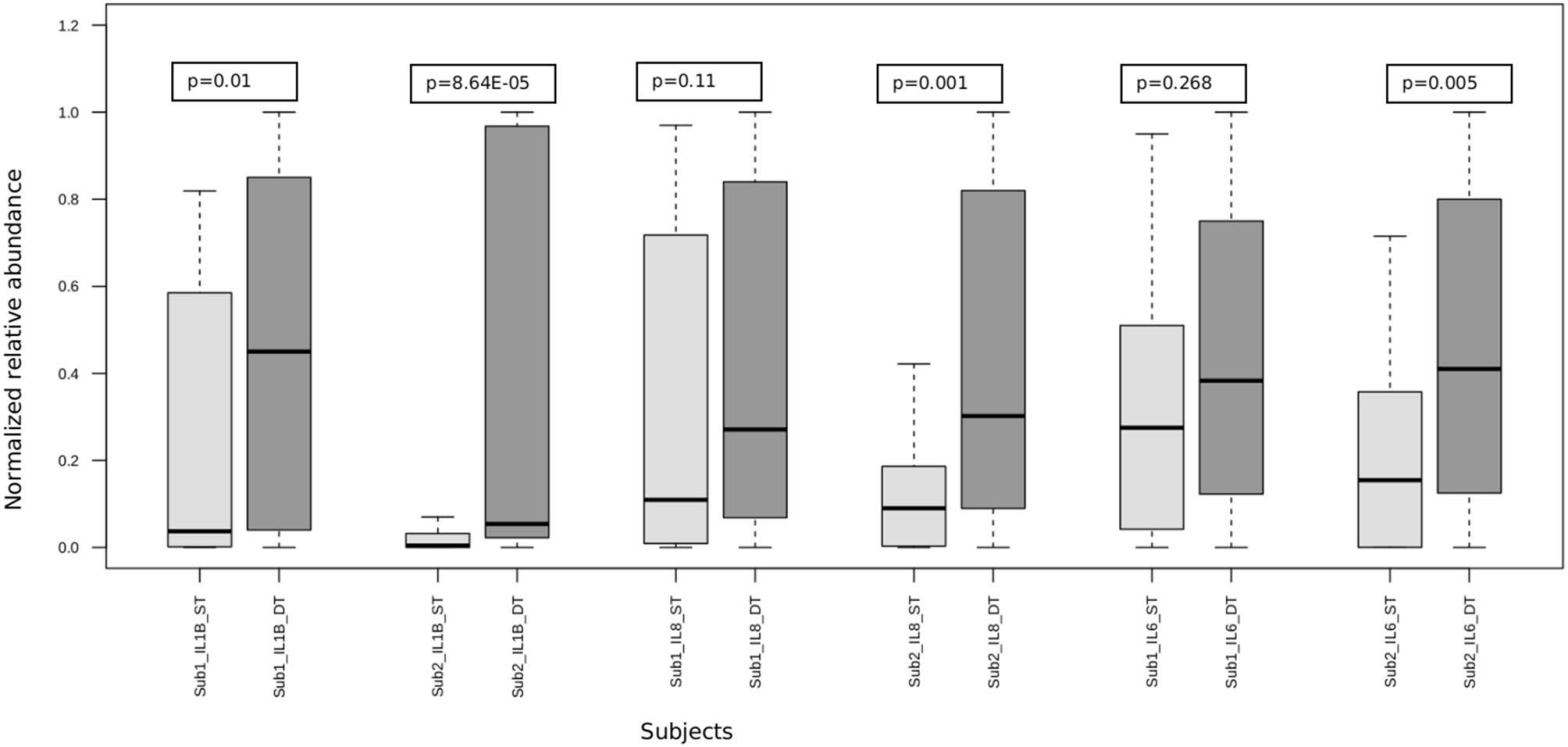
Differences in the normalized cytokine levels at same and different time points for 2 subjects over 3 days. Here, three cytokines were studied, IL1β, IL-8 and IL-6. Each boxplot represents a subject’s cytokine level at same (ST) or different (DT) time points. Mann–Whitney test was used to determine if the values across the groups (ST and DT) differ and the corresponding p-value obtained have been written on the top of each plot. All the differences were statistically significant except at Subject 1 IL-8 and IL-6.

**Figure 9:**
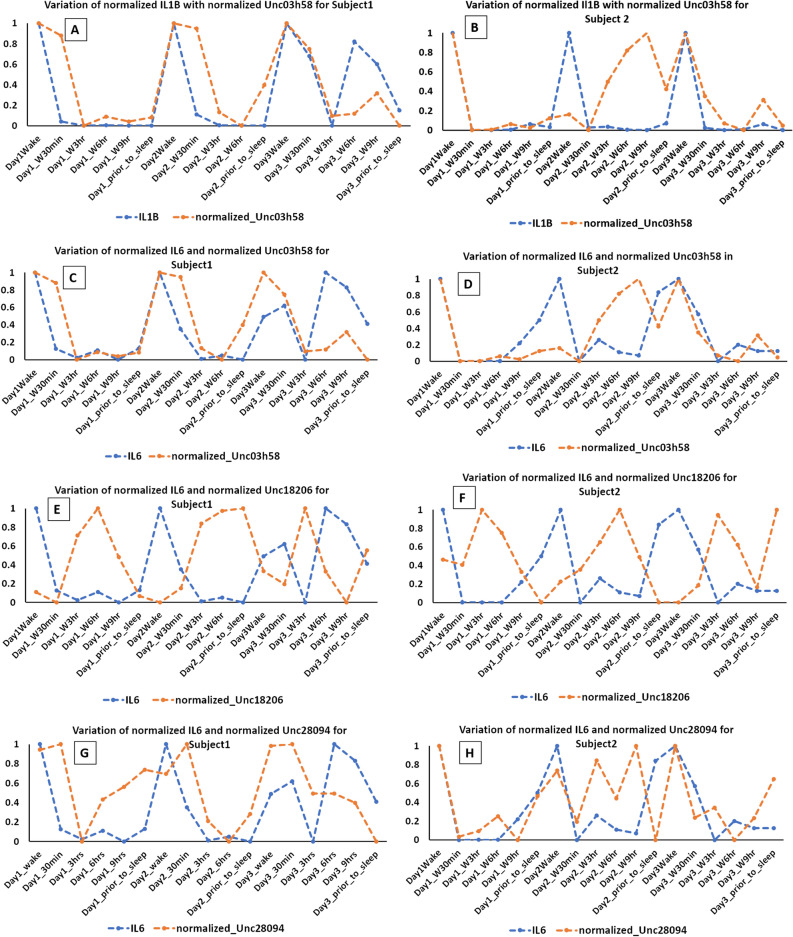
Plots showing association of OTU and normalized cytokine level in two subjects over three days. Figures for Subjects 1 and 2, respectively: (**A**,**B**) Positive correlation and periodicity between IL-1β and Unc03h58 (*Prevotella*), (**C**,**D**) Positive correlation and periodicity between IL6 and Unc03h58 (*Prevotella*), (**E**,**F**) Negative correlation and periodicity between IL6 and Unc18206 (*Neisseria*), (**G**,**H**) Positive correlation and periodicity between IL6 and Unc28094 (*Porphyromonas*).

For the two subjects with samples collected over three days, alpha diversity indices did not vary significantly at any of the time points (Fig. 7) as seen with the intraday analysis (Fig. 1). To examine microbiome and inflammatory oscillation patterns over three days, we performed the periodicity discrimination method (PDM) as described in Materials and Methods. The differences in the normalized concentrations of the cytokines were found to be considerably less for the same time points than at different time points (Fig. 8). Except for IL-6 and -8 for Subject 1, all differences were statistically significant. To check for periodicity of microbiome and OTUs, our correlation analysis identified four such OTU-cytokine pairs which are listed in Supplementary Table S3. The corresponding plots are displayed in Fig. 9. The OTU Unc03h58 (*Prevotella*) showed significant periodicity to IL-1β and IL-6 in both subjects, while Unc28094 (representing *Porphyromonas*) displayed periodic fluctuations with IL-6 levels. In contrast, Unc18206 (representing *Neisseria*) displayed inverse periodicity with the fluctuations of IL-6. Taken together, these findings revealed a periodicity of both the oral microbiome (OTUs) and inflammation (cytokine levels) with identifiable patterns between IL-1β and *Prevotella*, and IL-6 with *Prevotella*, *Neisseria* and *Porphyromonas*.

## Discussion

The novelty of our study is that both the salivary inflammatory profile and microbiome was simultaneously determined longitudinally within a 24-h period and across three days. In addition, this study is the first to our knowledge to examine the oscillatory association between bacterial diversity and composition with oral inflammation. In addition, we evaluated the association between cytokine concentration and bacterial OTU’s using a negative binomial mixed model regression. In summary, our study demonstrated that both oral inflammation (as measured by salivary IL-6, IL-8 and IL-1β) and salivary microbiome follow an oscillatory pattern in association with one another. Furthermore, certain bacterial genera, such as *Prevotella*, *Neisseria* and *Porphyromonas* demonstrated significant periodicity with precise cytokines.

Within the analysis of the 24-h. time course among 12 individuals, alpha-diversity analysis revealed no significant changes in the bacterial richness of the salivary microbiome across subjects within or across time points. In contrast, cytokine concentrations were much higher at wake and typically waned during the day, most significantly with IL-1β. Significant changes in genus abundance were observed during the 24-h. time course for the genera *Leptotrichia*, *Prevotella*, *Rothia* and *Neisseria*. *Granulicatella*, which is known as a member of the healthy oral microbiome, was found to be negatively associated with the pro-inflammatory cytokine IL-8. Our identified inverse relationship between *Granulicatella* and IL-8 was also identified upon the exposure of smoking. This has been known to induce the production of IL-8 and a decreased level of *Granulicatella* in the oral cavity^33^. *Prevotella* was observed to be negatively associated with IL-8 but positively associated with IL-1β. While it is known that *Prevotella* promotes the production of pro-inflammatory cytokines^34^, this genus comprises more than 40 different and diverse species some of which might play a different role than the rest of the members. For example, conflicting results have been obtained for the role of *Prevotella* in the pathogenesis of rheumatoid arthritis^35^.

The periodicity we observed for the oral microbiome is consistent with the findings of Takayasu et al.^26^*.* They examined salivary 16S rRNA sequences from 6 healthy adults collected at 4-h intervals for three days and observed significant oscillations with a periodicity of approximately 24 h. The genera of *Prevotella, Gemella, Streptococcus* and *Haemophilus* demonstrated significant circadian periodicity. This was highly like our findings where we also observed periodicity with *Prevotella.* However, *Neisseria* and *Porphyromonas* periodicity was not observed in their analysis. Explanations for different findings may be explained by variations in sample size or sequencing methods. For example, our analysis was based on the 16S rRNA V4 region, whereas their examination was evaluated on the V1–V2 region. Variation in 16S rRNA primer usage can result in differences with observed phylogenetic abundance and may explain the potential over or underestimation of certain populations^36,37^.

Furthermore, our findings revealed significant periodicity of IL-1β with a general trend for IL-8 and IL-6 in the saliva of healthy adults consistent with other examinations of diurnal variation or circadian rhythm of inflammatory markers in the saliva. Prior studies have revealed that detectable salivary inflammatory biomarkers do show diurnal variations as has been demonstrated with IL-6, MCP-1, IL-1β, and C-reactive protein^38–40^.

Our most significant and novel results revealed significant associations between bacterial populations *Prevotella* and *Ruminococcaceae* with IL-1β concentration while *Prevotella* and *Granulicatella* were associated with IL-8. Our findings have also identified periodicity between IL-1β and *Prevotella*, and IL-6 with *Prevotella*, *Neisseria* and *Porphyromonas*. To our knowledge, this relationship has not previously been reported. The biological explanation or relationship for the above observed associations have yet to be fully determined with exception that they both are regulated by circadian oscillation. One plausible biological explanation for the relationship between the microbiome and inflammation periodicity may be quorum sensing. Fteita et al*.* examined the cytokine response of human gingival keratinocytes (HMK) exposed to the cell extract of three different *Prevotella* species^41^. Both IL-6 and IL-8 secretion was increased from HMK cells incubated with estradiol and cell extract of *P. intermedia* and *P. nigrescens*. Further studies have shown that IL-6 expression is increased from human dental pulp cell cultures stimulated with *Prevotella intermedia* lipopolysaccharide^42^. In addition, *Neisseri*a has been demonstrated to suppress IL-6 production and our associations have indeed revealed that abundance of oral *Neisseria* is inversely correlated to IL-6 concentration^43^. Taken together, numerous studies have clearly demonstrated that oral bacteria utilize cytokines to sense the host environment and there is a dynamic interplay between both. Feeding time could be the important connecting link between the oral microbiome and the levels of cytokines in the saliva. It has been previously speculated that the need to metabolize various substrates post a meal might affect the bacterial abundances^44^. Also, the exposure of oral bacteria to lysozyme during feeding could destroy a part of the bacterial population. Fasting has also been found to be associated with oral cytokine levels^45^. Although in this study, the participants did not follow a fast, feeding habit such as caloric restriction is known to affect cytokine levels^46^.

The variation observed for both cytokine levels and some investigated bacteria particularly at wake are concordant with the limited previous publications that have also examined diurnal rhythms or periodicity. Some of the reasons for this may be attributed to standard biological variation (i.e. age, gender, diet, or stress) that may be observed with typical *in-vivo* measurements. In addition, small sample size may have also contributed to this increased variability at wake. For example, the level of Actinobacteria (Fig. 4) was found to decrease over the day in a previous study although it was based on the gut microbiome in mice model^47^. Similarly, we too observed that the Bacteroidetes level was highest in the morning and at night in comparison to other times of the day as observed previously on mouse models^48^. In our results at the genera level too (Fig. 5) we observed consistent patterns where the levels of Prevotella was high in the morning while that of Streptococcus was observed to be higher in the evening^26^.

With regard to the variation observed for IL-6 at wake, this is concordant with the limited reports examining diurnal variation of oral cytokines. Kessler et al*.* evaluated if saliva samples could be employed to evaluate the effect of timing of carbohydrate and fat intake on metabolic rhythms^38^. Similar to our results they found the highest levels of inflammation at wake with lowest levels mid-day. Furthermore, they also found a large variation in salivary IL-6, particularly at wake. In their study, the authors suggested that the small sample size (n = 14) may have explained this biological variation. Standard biological variation at wake coupled with small sample size may have attributed to our corresponding findings as well.

Diet is certainly an important parameter that must be considered in oral inflammatory and microbiome studies. Interestingly, the study by Kessler et al. also demonstrated that the time of consumption for carbohydrate and fat intake has little to no effect on both oral metabolic and inflammatory biomarkers examined in the saliva^38^. However, long-term dietary patterns do influence the oral microbial community. Hansen et al*.* demonstrated that compositional differences within the salivary microbiome of vegans and omnivores is present at taxonomic levels below phylum^49^. Similar findings have been also identified within the gut microbiome^50^. All but one of our participants were omnivores, and therefore our observed microbiome and inflammatory periodicity are likely not majorly impacted by chronic diet patterns. However, future studies should certainly include both dietary records and habits.

One of the major limitations of our study is the lack of Whole Genome Sequencing (WGS). WGS would have provided further refined data to reveal the specific species populations particularly associated with inflammatory levels and oscillatory patterns. Another limitation is the number of inflammatory markers examined. Additional markers of inflammation such as chemokines or markers of tissue destruction known as matrix-metalloproteinases (MMP’s) or even inhibitors of MMP’s such as TIMPS would have increased further examination on the associations with the microbiome. Measurement of anti-inflammatory cytokines such as IL-10 would have also been useful to provide a comprehensive examination determining the overall balance between oral pro- and anti-inflammatory cytokines. Additionally, the sample size is small, with only 12, mostly White, non-Hispanic adults providing samples over a 24-h period and just 2 providing samples over three days. Larger studies with a more heterogeneous population are needed to confirm these findings. We also did not have data on potential mediators of these relationships, such as diet or objective measures of oral health, albeit we did incorporate a self-reported assessment.

Taken together, our study has demonstrated that both the oral microbiome and inflammation demonstrate measurable oscillatory patterns and there are distinct associations between both. These results have implications for studies that will utilize oral biomarkers for future clinical utility and therefore indicate that normalization for time of collection may account for daily fluctuations. One suggestion for future studies would be to employ multiple collection and temporal harmonization of samples to examine and account for inflammatory and microbiome periodicity. This would greatly improve and enhance the ability to discriminate biomarkers or changes in microbiome with respect to association with a clinical disease or measure.

## Methods

### Participants

A longitudinal observational study of 12 healthy individuals recruited from the University of South Florida, aged 18 or older was performed to measure diurnal variation in both the oral microbiome and inflammation over time. None reported infections, immune disorders, or oral/dental health problems including salivary gland disorders, and none used any medications or dietary supplements. The study was reviewed and approved by the University of South Florida Institutional Review Board under protocol Pro00018592. The authors confirm that all research was performed in accordance with relevant guidelines and regulations. Informed consent was obtained from all participants.

### Saliva collection

Following explanation of study and protocol, voluntary participants were initially trained on collection and subsequently asked to passively drool into a 2 mL collection vial with an attached salivary collection aid (Saliva Biol LLC No. 61/524096 patent pending) during the recommended time points (see Saliva collection timings). Unstimulated whole saliva often correlates to systemic clinical conditions more accurately than stimulated saliva, since materials used to stimulate flow may change salivary composition^51^. The collection vials contained a protease and phosphatase inhibitor cocktail (EDTA-free Thermo Scientific Halt Thermos Fisher Scientific, Rockford, IL, USA) at 1 × to assist in cytokine preservation of the saliva sample. Sampling was typically performed at the participants’ homes or within the laboratory with supplied and labeled tubes with instructions. Following collection, saliva samples were frozen in the participants’ home freezers and transported in insulated containers with ice-packs to laboratory following completion of the collected samples. The sample was centrifuged for 5 min at 4000 rpm in a refrigerated centrifuge set at 4 °C. The saliva sample was then distributed into 100 µL and 350 µL aliquots and stored immediately at − 80 °C for further multiplexing and microbiome analysis. Commercially received saliva (Innovative Research, Novi, MI, USA) was also aliquoted and stored frozen to be employed in each multiplexing batch for the purpose of quality control.

### Saliva collection timings

Participants were instructed to provide saliva samples 6 times in a day (at wake, + 30 min from wake, + 3 h from wake, + 6 h from wake, + 9 h from wake, and bedtime). The participants were not requested to change their sleep/wake schedule for this study. Two participants were asked to repeat this sampling procedure on 3 consecutive days to examine periodicity. Participants were instructed to refrain from eating, drinking, heavy exercising, or brushing their teeth for 30 min after waking and for 1 h prior to each sampling time. Written instructions regarding the saliva collection protocol were provided. In addition, participants completed an oral health questionnaire. This questionnaire consisted of two questions regarding gingival condition: (1) Compared to others your age, how would you rate the current condition of your gums: poor, fair, good, excellent, and (2) Do you have a loose tooth? These questions were previously shown to be correlated with clinically determined periodontal health^52^.

### Measurement of inflammation

Cytokine levels were determined using a multiplexed bead immunoassay and measured with a Luminex MAGPIX instrument (Luminex, Austin, TX, USA) as previously detailed^6^. The cytokines of IL-1β, IL-6, and IL-8 were measured using the high sensitivity human cytokine magnetic bead assay (EMD Millipore, Cat No. HSCYTMAG-28SK, Billerica, MA, USA) following manufacturer’s instructions. Each assay was analyzed on the Luminex MAGPIX instrument to measure inflammatory concentrations followed by a 5-parameter logistic curve-fitting method from a standard curve of each respective analyte. Saliva samples were normalized by equivalent volume of commercially supplied dilution buffer and examined in duplicate per each assay run. The high sensitivity human cytokine magnetic bead kit provides a minimum detectable concentration of IL-1β (0.12 pg/mL), IL-6 (0.13 pg/mL) and IL-8 (0.12 pg/mL)^6^. Quality control samples of saliva from healthy volunteers were included in each assay run to account for any potential intra or inter-assay variability. Concentration was calculated by the StatLIA Immunoassay Analysis software (Brendan Technologies), by measuring the true limits of detection for an assay by mathematically determining what the empirical Minimum Detectable Concentration (MinDC) would be if an infinite number of standard concentrations were run for the assay under the same conditions.

In addition, salivary cotinine levels were measured utilizing a commercial ELISA (Salimetrics, Catalog Number 1-2002, Carlsbad, CA, USA) according to manufacturer’s instructions. Cotinine levels (ng/mL) were determined from 20 µL of saliva and measured in duplicate. A measurement greater than 25 ng/mL indicated that the subject was exposed to second-hand smoke or was smoking on the day of collection.

### 16S microbiome analysis

A 350 µl aliquot from the originally collected saliva sample was shipped on dry ice to The Alkek Center for Metagenomics and Microbiome Research at Baylor College of Medicine for microbiome analysis. DNA was extracted using the Mo BIO PowerMag Microbiome kit (Qiagen, Valencia CA). The resulting heterogeneous pool of bacterial genomic DNA was used as the template for PCR amplification with the primer pair of 515F (5′-GTGCCAGCMGCCGCGGTAA-3′) and 806R (5′-GGACTACHVGGGTWTCTAAT-3′) that amplify the 16S V4 rRNA gene^53^. The resulting 16S rRNA PCR products were purified and quantified before being pooled and sequenced on the Illumina MiSeq platform (2 × 250 bp). Paired-end 16S amplicon sequencing reads were demultiplexed using ea-utils command line tools (https://code.google.com/p/ea-utils/), and clustered into operational taxonomic units (OTUs) using the UPARSE144 pipeline. Reads were quality-filtered using the UPARSE quality-filtering threshold of Emax = 1, at which the most probable number of base errors per read is zero for filtered reads. Filtered reads were trimmed to a fixed length, singletons removed, and clustered into de-novo OTUs, with simultaneous chimera filtering. Taxonomic classification of OTUs was performed against the Greengenes146 version 13.5 16S rDNA database. Alpha Diversity Analysis was performed to examine both bacterial richness and evenness temporally within the saliva samples with determination of Observed Operational Taxonomic Units (OTUs), Chao1 (estimator of diversity), and Shannon Diversity Index (richness and evenness).

### Statistical analyses

The statistical analyses were carried out with two approaches, (a) association testing between salivary microbiome and cytokine levels for the 12 subjects with six samples collected over a single day, and (b) identification of oscillation patterns as determined by the periodicity discrimination method (PDM) for the 2 subjects with samples collected over 3 days.

### Association between salivary microbiome and cytokine levels

The association between the cytokines (dependent variable) and the salivary microbiome OTUs (independent variable) were tested using negative binomial mixed model regression with a random intercept using the R (https://www.r-project.org/) package NBZIMM^54^. In the original paper, the authors had used the OTU counts as the outcome for the negative binomial regression. However, considering the high variability of the cytokine data in our samples, we found that the requirements for applying negative binomial regression for cytokine as outcome was justified. For this purpose, the one-day data (six time points) from 12 subjects were utilized. OTUs present in less than 5% of the samples and in less than 50% of the subjects were discarded to ensure that results weren’t skewed by rare OTUs present in a minority of subjects. Models were adjusted for the age of participants, gender, BMI, gum status and the time of sample collection.

### Identification of oscillation patterns

The periodicity was based on the microbiome and cytokine data from two subjects collected over a period of three days. To test the periodicity of the cytokine levels, a normalization method was applied as described previously^26^. Briefly, we employed the formula:

$$\varvec{d_{norm} } = \frac{\varvec{d_n } - {\varvec{d_{min}}}}{{\varvec{d_{max} } - \varvec{ d_{min}}}}$$

to calculate the normalized levels of IL1β, IL-8 and IL-6 for each day in each subject. The term d_norm_ indicates the normalized level at a given time point, d_n_ denotes the reading at a time point. d_max_ and d_min_ are the maximum and minimum readings for the day for a given subject. To investigate if the variation in daily cytokine level follows a pattern, the normalized values were compared at the same time of the day (six times as mentioned earlier) to that of the different times over three days (all readings except the same time point). We also investigated if the alpha diversity varied significantly among the two subjects over the three-day period by calculating observed OTUs, Chao1 and Shannon Diversity Index.

In addition, we examined if any OTU (generally representing a bacterial genus) followed similar patterns of periodicity and whether it was correlated to the cytokine levels in the samples. First, the top 30 most abundant OTUs were identified and their correlation with the cytokine levels for each subject was tested separately in R using the stats package (R core team). The OTUs which displayed significant associations (positive and negative) were normalized in a similar method as described previously. Only OTUs which showed similar significant correlation in both subjects are reported.

## Supplementary information

Supplementary Tables.

## Data Availability

The datasets generated during and/or analyzed during the current study are available from the corresponding author on reasonable request.
