# Supplementary material for: Linking the oral microbiome and salivary cytokine abundance to circadian oscillations
Source: Sci Rep. 2021 Jan 29;11:2658. doi: 10.1038/s41598-021-81420-3 (PMC7846843; doi:10.1038/s41598-021-81420-3)
Supplement: Supplementary file 1 — Supplementary Tables. [file 41598_2021_81420_MOESM1_ESM.pdf]

Linking the oral microbiome and salivary cytokine abundance to circadian oscillations

(Supplementary information)

Anujit Sarkar[1], Melanie N. Kuehl[2†], Amy C. Alman[1] and Brant R. Burkhardt[2*]

[1]College of Public Health, University of South Florida, Tampa, FL, USA 33612.

[2]Department of Cell Biology, Microbiology and Molecular Biology, University of South Florida, Tampa, FL, USA 33620.

[†] Present address: IPS Labs, 1 Harvard Way, Hillsborough Township, NJ 08844

*** Corresponding author:** Brant R. Burkhardt

Please submit correspondence to: bburkhardt@usf.edu

Supplementary Table S1. Saliva collection times of all 12 subjects.

Subject #	Sample ID	Time of Collection	Relative Time of Collection Post-Wake
**001**	001 A	7:02 AM	Wake
	001 B	7:32 AM	Wake + 30 min.
	001 C	10:33 AM	Wake + 3 hrs.
	001 D	1:33 PM	Wake + 6 hrs.
	001 E	4:28 PM	Wake + 9 hours
	001 F	11:02 PM	Prior to Sleep
Day 2	001 A	7:29 AM	Wake
	001 B	8:00 AM	Wake + 30 min.
	001 C	11:02 AM	Wake + 3 hrs.
	001 D	2:02 PM	Wake + 6 hrs.
	001 E	5:00 PM	Wake + 9 hours
	001 F	12:21 AM	Prior to Sleep
Day 3	001 A	9:18 AM	Wake
	001 B	9:50 AM	Wake + 30 min.
	001 C	12:50PM	Wake + 3 hrs.
	001 D	3:50 PM	Wake + 6 hrs.
	001 E	6:40 PM	Wake + 9 hours
	001 F	12:00 AM	Prior to Sleep
**002**	002 A	6:35 AM	Wake
	002 B	7:05 AM	Wake + 30 min.
	002 C	9:34 AM	Wake + 3 hrs.
	002 D	12:32 PM	Wake + 6 hrs.
	002 E	3:37 PM	Wake + 9 hours
	002 F	10:31 PM	Prior to Sleep
Day 2	002 A	6:20 AM	Wake
	002 B	6:50 AM	Wake + 30 min.
	002 C	9:24 AM	Wake + 3 hrs.
	002 D	12:36 PM	Wake + 6 hrs.
	002 E	3:36 PM	Wake + 9 hours

	002 F	10:52 PM	Prior to Sleep
Day 3	002 A	6:20 AM	Wake
	002 B	6:50 AM	Wake + 30 min.
	002 C	9:36 AM	Wake + 3 hrs.
	002 D	12:34 PM	Wake + 6 hrs.
	002 E	3:31 PM	Wake + 9 hours
	002 F	10:48 PM	Prior to Sleep
**003**	003 A	6:00 AM	Wake
	003 B	6:30 AM	Wake + 30 min.
	003 C	9:00 AM	Wake + 3 hrs.
	003 D	12:00 PM	Wake + 6 hrs.
	003 E	3:00 PM	Wake + 9 hours
	003 F	12:00 AM	Prior to Sleep
**004**	004 A	6:35 AM	Wake
	004 B	7:10 AM	Wake + 30 min.
	004 C	10:11 AM	Wake + 3 hrs.
	004 D	1:13 PM	Wake + 6 hrs.
	004 E	4:16 PM	Wake + 9 hours
	004 F	11:42 PM	Prior to Sleep
**005**	005 A	9:30 AM	Wake
	005 B	10:00 AM	Wake + 30 min.
	005 C	1:07 PM	Wake + 3 hrs.
	005 D	4:07 PM	Wake + 6 hrs.
	005 E	7:05 PM	Wake + 9 hours
	005 F	10:06 PM	Prior to Sleep
**006**	006 A	9:55 AM	Wake
	006 B	10:25 AM	Wake + 30 min.
	006 C	12:55 PM	Wake + 3 hrs.
	006 D	3:55 PM	Wake + 6 hrs.
	006 E	6:55 PM	Wake + 9 hours
	006 F	2:05 AM	Prior to Sleep

**007**	007 A	9:00 AM	Wake
	007 B	9:30 AM	Wake + 30 min.
	007 C	12:00 PM	Wake + 3 hrs.
	007 D	3:00 PM	Wake + 6 hrs.
	007 E	6:00 PM	Wake + 9 hours
	007 F	11:30 PM	Prior to Sleep
**008**	008 A	6:55 AM	Wake
	008 B	7:30 AM	Wake + 30 min.
	008 C	11:15 AM	Wake + 3 hrs.
	008 D	2:30 PM	Wake + 6 hrs.
	008 E	5:30 PM	Wake + 9 hours
	008 F	1:00 AM	Prior to Sleep
**009**	009 A	8:00 AM	Wake
	009 B	8:30 AM	Wake + 30 min.
	009 C	11:30 AM	Wake + 3 hrs.
	009 D	2:30 PM	Wake + 6 hrs.
	009 E	5:28 PM	Wake + 9 hours
	009 F	1:00 AM	Prior to Sleep
**010**	010 A	8:00 AM	Wake
	010 B	8:31 AM	Wake + 30 min.
	010 C	11:35 AM	Wake + 3 hrs.
	010 D	2:35 PM	Wake + 6 hrs.
	010 E	5:35 PM	Wake + 9 hours
	010 F	1:07 AM	Prior to Sleep
**011**	011 A	6:10 AM	Wake
	011 B	6:40 AM	Wake + 30 min.
	011 C	9:10 AM	Wake + 3 hrs.
	011 D	12:10 PM	Wake + 6 hrs.
	011 E	3:10 PM	Wake + 9 hours
	011 F	11:00 PM	Prior to Sleep
**012**	012 A	7:45 AM	Wake
	012 B	8:15 AM	Wake + 30 min.

	012 C	10:45 AM	Wake + 3 hrs.
	012 D	1:45 PM	Wake + 6 hrs.
	012 E	4:45 PM	Wake + 9 hours
	012 F	11:00 PM	Prior to Sleep
**Average Wake Time*†**	7:47 A.M. ± 1.3		
**Average Time to Bed*†**	12:17 A.M. ± 1.3		

*Across 12 participants and not including Days 2 and 3 from Participants 1 and 2.
†Data presented as mean time ± S.D.

Supplementary Table S2: Association of OTUs and cytokine levels for 12 subjects based on mixed model regression (Random intercept model).

Cytokine	Variable	Classification	Value	Std. error	t-value	P-value
IL-8	Unc95609	Aggregatibacter	0.0146	0.007550	1.931	0.089
	UncPrev6	Prevotella6	-0.0075	0.003104	-2.416	0.042
	UncGran4	Granulicatella	-0.0149	0.006105	-2.438	0.04
	Unc02022	Atopobium	0.0323	0.014331	2.255	0.054
IL-1β	Unc03h58	Prevotella	0.0065	0.0032	2.024	0.07
	Unc25687	Prevotella7	0.0012	0.0004	2.911	0.019
	Unc02zoe	Ruminococaceae	0.2797	0.1071	2.612	0.031

	StrThe42	Streptococcus	-0.0186	0.0054	-1.998	0.08
	SrBacte2	SR1	-0.0777	0.0213	-3.647	0.006

Supplementary Table S3. Significant correlations between cytokine levels and microbial OTUs based on 2 subjects 3-days data.

S.no.	Cytokine	OTU	Subject1_correlation coefficient	Subject1_pvalue	Subject2_correlation coefficient	Subject2_pvalue
1	IL1B	Unc03h58	0.63	0.007	0.71	0.0007
2	IL6	Unc03h58	0.56	0.02	0.72	0.0008
3	IL6	Unc18206	-0.46	0.06 (marginally significant)	-0.48	0.04
4	IL6	Unc28094	0.44	0.07 (marginally significant)	0.69	0.001
